# CLP1, a Novel Plant Homeo Domain Protein, Participates in Regulating Cellulase Gene Expression in the Filamentous Fungus *Trichoderma reesei*

**DOI:** 10.3389/fmicb.2019.01700

**Published:** 2019-08-06

**Authors:** Lei Wang, Renfei Yang, Yanli Cao, Fanglin Zheng, Xiangfeng Meng, Yaohua Zhong, Guanjun Chen, Weixin Zhang, Weifeng Liu

**Affiliations:** State Key Laboratory of Microbial Technology, Microbial Technology Institute, Shandong University, Qingdao, China

**Keywords:** *Trichoderma reesei*, cellulase gene, transcriptional regulator, CLP1, XYR1

## Abstract

The stringent regulatory network of cellulase gene expression in the filamentous fungus *Trichoderma reesei* involves multiple transcriptional regulators. However, identification and mechanistic investigation of these regulators are still insufficient. Here, we identified a novel transcriptional regulator, CLP1, a plant homeo domain (PHD) Protein that participates in regulating *T. reesei* cellulase gene expression. Phylogenetic analyses demonstrated that CLP1 homologs are widely distributed in filamentous fungi including *Trichoderma*, *Penicillium*, *Fusarium*, *Neurospora*, and *Aspergillus* species. We demonstrated that CLP1 is a nuclear protein and lack of CLP1 significantly impaired the induced expression of cellulase genes. ChIP experiments showed CLP1 binding to the cellulase gene promoters specifically under cellulose conditions and compromised XYR1 occupancy on the same promoters in the absence of CLP1 at the early induction stage. XYR1 overexpression fully rescued the defect in cellulase production but not the defect in conidia formation in the *clp1* null mutant. Further analysis showed that the PHD is required for the CLP1 appropriate subcellular localization as well as the induced cellulase gene expression and conidiation. Taken together, these data demonstrated an important role of CLP1 in the regulation of cellulase and xylanase gene expression in *T. reesei*.

## Introduction

Rapid and precise regulation of gene expression at the transcriptional level is essential for eukaryotic cells when confronted with environmental stresses and nutrition shift (Znameroski and Glass, 2013; Crawford and Pavitt, 2019). The filamentous fungus *Trichoderma reesei* is capable of efficiently initiating cellulase gene expression in response to insoluble cellulose and therefore has been applied for a long term for industrial cellulase production. *T. reesei* has also been established as an excellent model system for the investigation of mechanisms underlying induced gene expression (Druzhinina and Kubicek, 2017; Schmoll, 2018). Systematic characterization of the involved transcriptional factors would definitely contribute to understanding the whole regulatory network controlling cellulase gene expression in *T. reesei*.

Eukaryotic gene regulatory network involves multiple transcription factors and cofactors to integrate various environmental signals, and thus to exert regulatory effect on target genes by acting on their promoters (Tani et al., 2014). In *T. reesei*, several transcription factors involved in the regulation of cellulase gene expression have been identified, including the positive regulators XYR1 (Stricker et al., 2006), ACE3 (Häkkinen et al., 2014), CRZ1 (Chen et al., 2016), and ACE2 (Aro et al., 2001), and the negative regulators CRE1 (Strauss et al., 1995; Antoniêto et al., 2016), ACE1 (Saloheimo et al., 2000), and RCE1 (Cao et al., 2017). These identified factors belong to typical fungal transcription factors that bind to promoters *via* DNA-binding domains (Takashima et al., 1996; Saloheimo et al., 2000; Aro et al., 2001; Chen et al., 2016; Cao et al., 2017). Among others, XYR1 is the transcriptional activator crucial for cellulase and hemicellulose gene expression, although its precise acting mechanism is not yet clear (Stricker et al., 2006; Dos Santos Castro et al., 2016). Besides, chromatin status changes have been recently shown to occur in *T. reesei* for cellulase genes in context to the applied conditions (repressing/inducing) (Mello-de-Sousa et al., 2015). Specific chromatin modifiers such as SAGA and LAE1 have also been identified to participate in modulating cellulase gene expression (Seiboth et al., 2012; Xin et al., 2013). Despite these advances, other yet-to-be known factors are speculated to be involved in this intricate and stringent regulatory network controlling cellulase gene expression. Systematic identification and characterization of these regulatory factors would undoubtedly contribute to unveiling the elaborate mechanism of cellulase gene regulation in *T. reesei*.

Eukaryotic DNA is wrapped in proteins called histones to form nucleosomes. Recruitment of nucleosome modifiers such as histone acetyltransferases to specific promoter regions facilitates the activation of a gene packaged within chromatin, by loosening the chromatin structure to uncover binding sites for regulators and for the transcriptional machinery (Mizzen and Allis, 1998; Church and Fleming, 2018). Proteins containing plant homeo domain (PHD) typified with a well-conserved Zn finger motif of the (Cys4)-His-(Cys3) type have been reported to be involved in chromatin-mediated transcription regulation (Aasland et al., 1995; Sanchez and Zhou, 2011; Bhushan et al., 2018). One such PHD finger-containing protein of *Saccharomyces cerevisiae*, CTI6, has been shown to participate in securing the rapid *GAL1* transcriptional activation *via* acting in concert with the histone acetyltransferase-containing complex SAGA to alleviate Cyc8-Tup1-mediated repression (Papamichos-Chronakis et al., 2002; Han and Emr, 2011). On the other hand, CTI6 has been found to play important roles in regulating *PHO5* gene in association with Rpd3-Sin3 histone deacetylase complex involved in transcriptional repression (Wang et al., 2011). Moreover, CTI6 is also required for the activation of several other genes including *SUC2* involved in sucrose uptake (Papamichos-Chronakis et al., 2002), *ICL1* and *FBP1* involved in gluconeogenesis (Han and Emr, 2013), the hypoxic gene *ANB1* (Papamichos-Chronakis et al., 2002), and *STE6* involved in cell-type-specific expression (Vidal and Gaber, 1991; Puig et al., 2004). Despite this knowledge regarding *S. cerevisiae* CTI6, evidence about the function of other fungal CTI6-like proteins in regulating gene transcription is still lacking.

In this study, we searched the genome of *T. reesei* with yeast CTI6 as the query and retrieved a PHD finger-containing protein, CLP1. We found that CLP1 plays important roles in cellulase gene expression and conidia formation. The defect in cellulase expression but not conidia formation in the *clp1* null mutant was fully rescued by XYR1 overexpression. We also showed that CLP1 is localized in the nucleus and recruited to cellulase gene promoters in the presence of cellulose. Furthermore, mutation analyses demonstrated that the integrity of the PHD is essential for the appropriate nuclear localization and function of CLP1.

## Materials and Methods

### Strains, Media, and Culture Conditions

*T. reesei* TU-6 (ATCC MYA-256), a uridine auxotroph of *T. reesei*, was used throughout this work as the parental strain. A *pyr4*-complemented TU-6 strain named TU6-RP (Cao et al., 2017), wherein the *pyr4* gene was homologously integrated into its native locus, was used as the control strain. For analyses of (hemi)cellulase production, *T. reesei* cells were pre-cultured at 30°C for 48 h on a rotary shaker (200 rpm) in 1-L Erlenmeyer flasks containing 250 ml of Mandels-Andreotti (MA) medium supplemented with 1% (v/v) glycerol. The mycelia were then harvested by filtration and washed twice with medium containing no carbon source. Equal amounts of mycelia were transferred to fresh MA medium containing 10 g/L (w/v) Avicel or 5 g/L (w/v) xylan as the sole carbon source, and the incubation was continued for the indicated time periods (Wang et al., 2018). Uridine at a final concentration of 10 mM was added for *T. reesei* cultivation when necessary. *T. reesei* was grown in daylight and dark cycles for both plate and liquid culture. *Escherichia coli* DH5a (TSINGKE, Beijing, China) was used for routine plasmid construction.

### Plasmids and Recombinant *T. reesei* Strains Construction

To delete the *clp1* gene, two approximately 2.0 kb of *clp1* up- and downstream noncoding sequences were amplified from *T. reesei* genomic DNA and ligated into pDonor-*pyr4* (Zhang et al., 2013) *via* BP cloning to yield the deletion vector pDonor-*pyr4-clp1*, which was used to transform *T. reesei* TU-6 after linearization with I-*Sce*I to result in the Δ*clp1* strain. To construct the plasmid P*_gpd_*-*clp1*-T*_trpC_*, the full-length *clp1* was amplified from the genomic DNA of *T. reesei* and inserted into the P*_gpd_*-T*_trpC_*-*hph* vector, which was generated by inserting the *gpd* promoter and T*_trpC_* terminator amplified from the genomic DNA of *A. nidulans* into the pUC19-*hph* plasmid (Zhou et al., 2012). The PHD mutant of *clp1* with two mutations of C145A and H150A and the UIM (ubiquitin interacting motif) deletion mutant of *clp1* were obtained by overlap-extension PCR (Heckman and Pease, 2007; Zhang et al., 2015), and similarly inserted into the P*_gpd_*-T*_trpC_*-*hph* plasmid to construct P*_gpd_*-*clp1*_PHDM-T*_trpC_* and P*_gpd_*-*clp1*_ΔUIM-T*_trpC_*, respectively. These three plasmids P*_gpd_*-*clp1*-T*_trpC_*, P*_gpd_*-*clp1*_PHDM-T*_trpC_*, and P*_gpd_*-*clp1*_ΔUIM-T*_trpC_* were transformed to the Δ*clp1* strain to obtain the Re*clp1*, Re*clp1*_PHDM, and Re*clp1*_ΔUIM strains, respectively. To express the mCherry-tagged histone H2B, a reference nuclear protein, in *T. reesei*, the *xyr1* sequence of the P*_tcu1_*-*mCherry*-*xyr1* (Zheng et al., 2017) vector was replaced by the H2B coding sequence to generate P*_tcu1_*-*mCherry*-*h2b*, which was then transformed into TU-6, resulting in the *mCherry*-*h2b* strain. To determine the subcellular localization of the native CLP1, the coding sequence of CLP1 fused with a C-terminal GFP was inserted into pMDP*_tcu1_*-T*_trpC_* (Lv et al., 2015) to result in P*_tcu1_*-*clp1*-*gfp*-T*_trpC_*, which was subsequently co-transformed with pRLMex-30 (Mach et al., 1994) into *mCherry*-*h2b* to generate the *mCherry*-*h2b*&*clp1*-*gfp* strain. The same strategy was used for construction of *mCherry*-*h2b*&*clp1*_PHDM-*gfp* and *mCherry*-*h2b*&*clp1*_ΔUIM-*gfp* strains, which are used for the determination of the localization of the two CLP1 mutants, respectively. To detect the recruitment of CLP1 on the cellulase gene promoters, the P*_tcu1_*-*clp1*-*gfp*-T*_trpC_* plasmid was co-transformed with pRLMex-30 into Δ*clp1*, to result in the P*_tcu1_*-*clp1*-*gfp* strain. To overexpress ACE3 in the Δ*clp1* strain, the *ace3* coding sequence was amplified from cDNA and inserted into pMDP*_tcu1_*-T*_trpC_* (Lv et al., 2015) to generate pMDP*_tcu1_*-*ace3*-T*_trpC_*. Then, pMDP*_tcu1_*-*ace3*-T*_trpC_* was co-transformed with pRLMex-30 into Δ*clp1* to result in the Δ*clp1*&OE*ace3* strain. Similarly, the pMDP*_tcu1_*-*xyr1*-T*_trpC_* (Lv et al., 2015) was co-transformed with pRLMex-30 into Δ*clp1* to result in the Δ*clp1*&OE*xyr1* strain. All the fungal transformations were performed as described by Penttilä et al. (1987). All the strains used are listed in Table 1 and verification of correct DNA integration in the mutant strains is shown in Supplementary Figure S1.

**Table 1:** Strains used in this study.

Strains	Description	Source
TU-6	ATCC MYA-256	Laboratory stock
TU6-RP	TU-6 complemented with *pyr4* expression cassette	(Cao et al., 2017)
Δ*clp1*	Deletion of *clp1* in TU-6	This study
Re*clp1*	Expression of CLP1 under a constitutive promoter in Δ*clp1*	This study
Re*clp1*_PHDM	Expression of CLP1 with two point mutations in PHD (C145A and H150A) in Δ*clp1*	This study
Re*clp1*_ΔUIM	Expression of CLP1 without UIM in Δ*clp1*	This study
Δ*clp1*&OE*xyr1*	Overexpression of XYR1 *in* Δ*clp1*	This study
Δ*clp1*&OE*ace3*	Overexpression of ACE3 in Δ*clp1*	This study
P*_tcu1_-clp1*-*gfp*	Expression of CLP1 tagged with C-terminal GFP in Δ*clp1*	This study
*mcherry*-*h2b*	Expression of histone H2B tagged with N-terminal mCherry in TU-6	This study
*mcherry*-*h2b*&*clp1*-*gfp*	Co-expression of CLP1-GFP and mCherry-H2B in TU-6	This study
*mcherry*-*h2b*& *clp1*_PHDM-*gfp*	Co-expression of CLP1 PHD mutant tagged with GFP (CLP1_PHDM-GFP) and mCherry-H2B in TU-6	This study
*mcherry*-*h2b*& *clp1*_ΔUIM-*gfp*	Co-expression of GFP-tagged CLP1 without the UIM (CLP1_ΔUIM-GFP) and mCherry-H2B in TU-6	This study

### Vegetative Growth and Conidiation Analyses

To analyze *T. reesei* vegetative growth, equal amounts of mycelia were inoculated on minimal media agar plates containing 1% (w/v) glucose, cellobiose, xylose, and arabinose incubated at 30°C for 2 days. To analyze *T. reesei* biomass on liquid MA medium with 1% glucose, equal amounts of mycelia were inoculated and the mycelia collected at growth intervals were dried and then weighed. For conidiation analysis, mycelia were inoculated on malt extract agar plates and incubated at 30°C for 5 days. The number of conidia was counted with a hemocytometer on an inverted optical microscope (Olympus, Tokyo, Japan).

### Quantitative RT-PCR

Total RNA was extracted using TRIzol reagent (Sangon, Shanghai, China) and purified using the TURBO DNA-free kit (Ambion, Austin, TX, USA) to remove gDNA according to the manufacturer’s instructions. Reverse transcription was carried out using the PrimeScript RT reagent Kit (Takara, Tokyo, Japan) according to the instructions. Quantitative PCR was performed on LightCycler 480 II (Roche, Basel, Switzerland). Amplification reactions were performed using the SYBR Green Supermix (Takara, Tokyo, Japan) according to the manufacturer’s instructions. Data analysis was performed using the comparative CT method (Schmittgen and Livak, 2008). The endogenous *actin* gene was used as the control for normalization.

### Enzymatic Activity and Protein Analysis

Extracellular cellulase activities were analyzed by measuring the amount of released *p*-nitrophenol using *p*-nitrophenyl-D-cellobioside (*p*NPC； Sigma) as substrate. The assays were performed in 200 μl of reaction mixtures containing 50 μl of diluted culture supernatant and 50 μl of substrate plus 100 μl of 50 mM sodium acetate buffer (pH 4.8) and were then incubated at 45°C for 30 min. One unit (U) of *p*NPCase activity is defined as the amount of enzyme releasing 1 μmol of *p*NP per minute. Xylanase activities were determined by measuring the amount of released xylose using xylan as substrate. Briefly, a reaction mixture containing 60 μl of diluted culture supernatant and 60 μl of beechwood xylan (5 g/L) dissolved in 50 mM sodium acetate buffer (pH 4.8) was incubated at 50°C for 15 min. The reducing sugar released in the mixture was determined using DNS method with xylose as the standard. One unit of enzyme activity was defined as the amount of enzyme capable of releasing 1 μmol of xylose per minute. SDS-PAGE was performed according to standard protocols as described by Laemmli (1970) with a 5% stacking gel and a 10% separating gel running at 160 V for 1.5 h. Equal amounts of culture supernatant relative to biomass were loaded for SDS-PAGE analysis of the extracellular proteins.

### Chromatin Immunoprecipitation

Chromatin immunoprecipitation (ChIP) assays were performed as described previously (Cao et al., 2017; Zheng et al., 2017). Briefly, the mycelia were incubated in MA medium containing 1% (w/v) glucose or Avicel plus 1% formaldehyde at 30°C for 10 min with shaking before the cross-linking was quenched *via* adding 25 ml of 1.25 M glycine. The mycelia were then collected; suspended in 50 mM HEPES lysis buffer at pH 7.5 plus 150 mM NaCl, 1 mM EDTA, 0.5% (v/v) Triton X-100, 0.1% (w/v) sodium deoxycholate, 0.1% (w/v) SDS, 1 mM PMSF (phenylmethanesulfonyl fluoride), 1 μg/ml leupeptin, and 1 μg/ml pepstatin; and broken with glass beads (0.45 mm). Chromatin DNA was further sonicated to obtain sheared DNA fragments with an average size of approximately 500 bp. Immunoprecipitation with the antibodies against XYR1 and GFP, respectively, was performed as previously described (Cao et al., 2017; Wang et al., 2019). Quantitative PCR was performed on the precipitated chromatin DNAs using the same procedure with qRT-PCR. Relative enrichment of the DNAs was calculated as a percentage of the input DNA.

### Subcellular Localization of CLP1 and Its Mutants

For visualization of the histone H2B, CLP1, and CLP1 mutants, spores of *mCherry*-*h2b*, *mCherry*-*h2b*&*clp1*-*gfp*, *mCherry*-*h2b*&*clp1*_PHDM-*gfp*, and *mCherry*-*h2b*&*clp1*_ΔUIM-*gfp* recombinant strains were inoculated into minimal medium containing 1% (w/v) glucose or Avicel, respectively. Mycelia after cultivation of 24 h (glucose) or 48 h (Avicel) were harvested for microscopic observation. Fluorescence was detected with a Nikon Eclipse 80i fluorescence microscope. The captured pictures of GFP and mCherry were merged using ImageJ2x software.

### Sequence Analysis

Amino acid sequences from *T. reesei* and other relevant species were obtained from the Uniprot^1^ databases. The phylogenetic tree was constructed by the maximum likelihood method using protein sequences aligned by the Muscle method based on the JTT model with MEGA6 (Hall, 2013; Tamura et al., 2013). Positions containing alignment gaps and missing data were partially deleted. Statistical confidence of the inferred phylogenetic relationships was assessed by performing 1,000 bootstrap replicates.

### Statistical Analysis

Statistical analysis was performed using the Student’s *t* test analysis. At least two to three biological replicates were performed for each analysis, and the results and errors are the mean and SD, respectively, of these replicates.

## Results

### Identification of the *clp1* Gene That Encodes a CTI6-Like Protein in *T. reesei*

**Figure 1:**
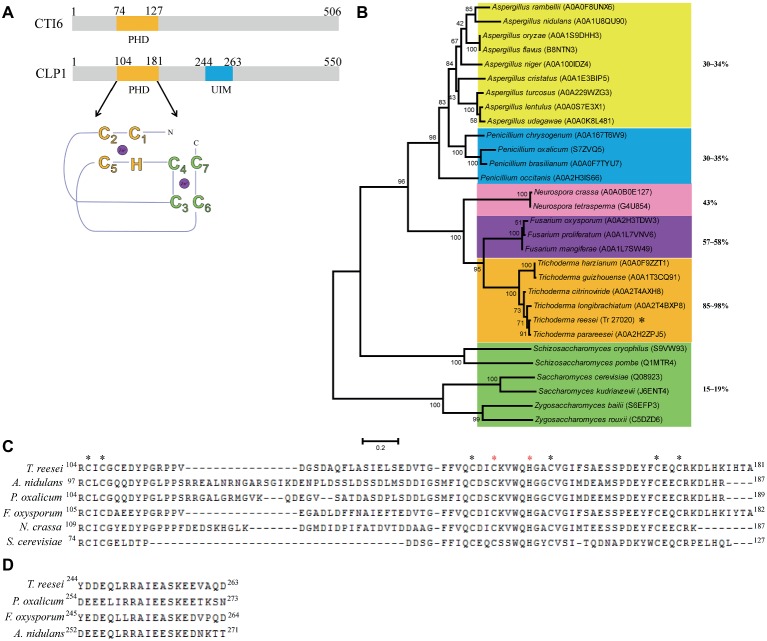
Sequence and phylogenetic analysis of CLP1 and its homologs. **(A)** Schematic diagram of CLP1 containing a conserved C4HC3 PHD finger and a UIM. The yeast CTI6 diagram is also shown. **(B)** Phylogenetic analysis of CLP1 and its homologs from *Trichoderma*, *Penicillium*, *Fusarium*, *Neurospora*, and *Aspergillus* species. The phylogenetic tree was generated with MEGA6. Numbers on the tree branches represent the bootstrap support calculated per 1,000 bootstrap replicates. The sequence identity between CLP1 and its homologs is indicated. **(C)** Sequence alignment of the PHD in CLP1 and its homologs. The conserved amino acid of C_4_HC_3_ motif is indicated by asterisks and the two residues (C145 and H150) to be mutagenized in this study are indicated with red asterisks. **(D)** Sequence alignment of the UIMs in CLP1 and its homologs.

*S. cerevisiae* PHD protein CTI6 has been implicated as an important factor in regulating the expression of several genes including *SUC2*, *GAL1*, *ANB1*, *ICL1*, and *FBP1* (Papamichos-Chronakis et al., 2002; Han and Emr, 2013). To analyze whether CTI6 homologous protein is present in the filamentous fungus *T. reesei*, we searched the *T. reesei* genome with CTI6 as a query and retrieved one gene (Trire2_27020 or TrireRUTC30_1_86967, hereafter named *clp1* for CTI6-like protein encoding gene). Although the overall amino acid sequence similarity and identity of CLP1 with that of CTI6 are only 29 and 19%, respectively, a well-conserved PHD finger of the (Cys_4_)-His-(Cys_3_) type was readily detected in CLP1 (residues 104–181) (Aasland et al., 1995; Bienz, 2006; Sanchez and Zhou, 2011). Moreover, an additional conserved UIM was found in CLP1 (residues 244–263) (Figure 1A; Hofmann and Falquet, 2001). The UIM (or LALAL motif) is a stretch of about 20 amino acid residues that is present in a variety of proteins either involved in ubiquitination and ubiquitin metabolism or known to interact with ubiquitin-like modifiers (Buchberger, 2002; Oldham et al., 2002; Polo et al., 2002). Unlike the PHD finger, the UIM present in CLP1 is not present in yeast CTI6 (Figure 1A).

To analyze the evolutionary relationship between CLP1 homologs from filamentous fungi and *Saccharomycotina/Saccharomyces* CTI6, phylogenetic analysis of CLP1 and its orthologs in other filamentous fungi including *Trichoderma*, *Penicillium*, *Fusarium, Neurospora*, and *Aspergillus* species as well as CTI6 orthologs from *Saccharomycotina*/*Saccharomyces* species was performed (Figure 1B). The results revealed that CTI6 homologs from *Saccharomycotina/Saccharomyces* species form a distinct cluster from those CLP1 homologs of filamentous fungi. Nevertheless, CLP1 is highly conserved in *Trichoderma* species with an overall sequence identity between 85 and 98%. Relatively higher sequence identity also exists among orthologs from *Fusarium* (~58%) and *Neurospora* (~43%), whereas lower sequence identity is found with those from the distantly related *Penicillium* (30–35%) and *Aspergillus* (30–34%) species, indicating that CLP1 orthologs are widely distributed in the above filamentous fungi although none of these orthologs have been characterized. Notably, the conserved PHD is present in all the above CLP1 orthologs (Figure 1C) whereas the UIM is observed in a vast majority of them except those in *Neurospora* species (Figure 1D).

### *T. reesei clp1* Mutant Exhibits a Conidiation Defect

**Figure 2:**
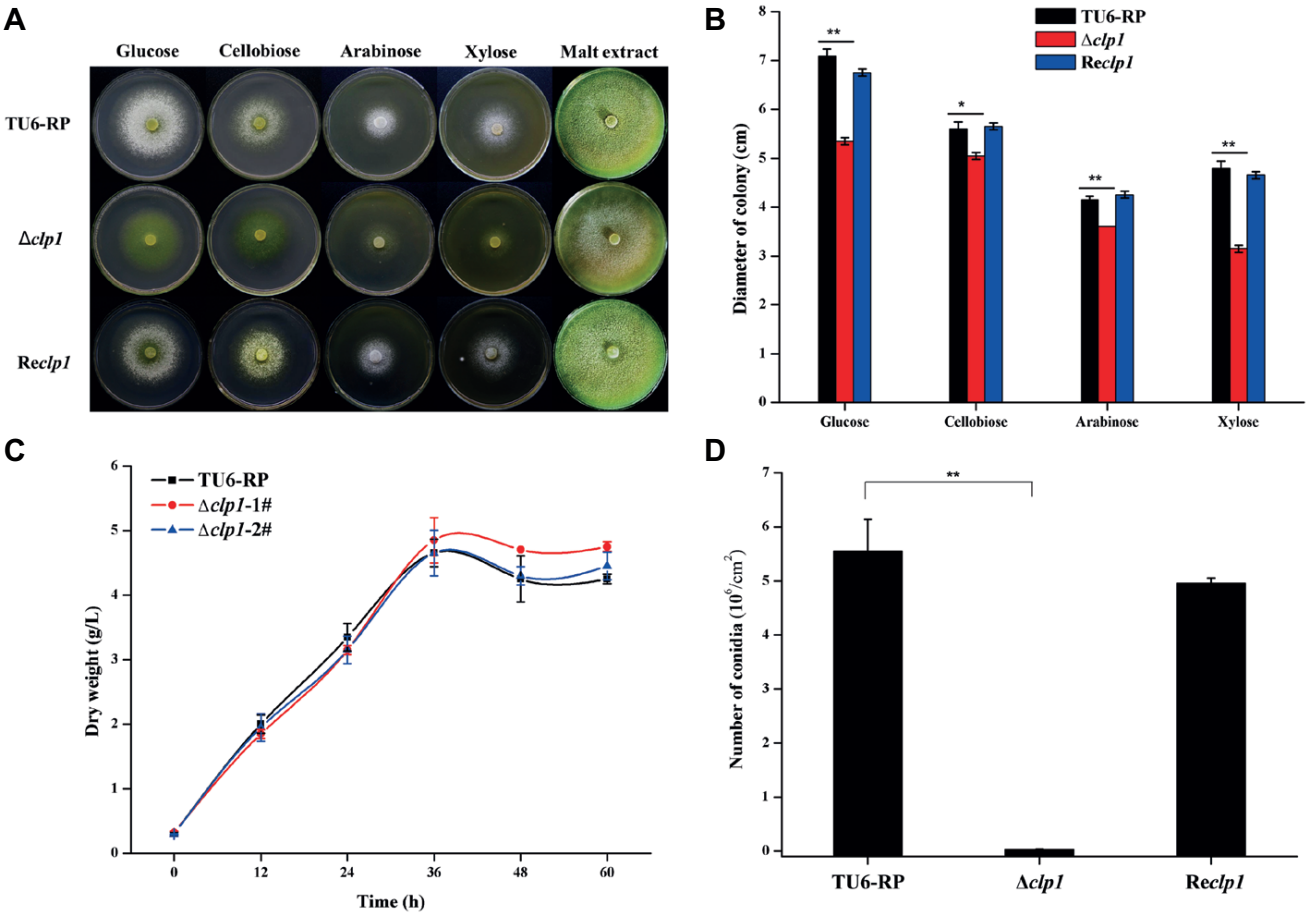
The effect of the absence of CLP1 on the vegetative growth and conidiation of *T. reesei*. **(A)** Colony growth of the Δ*clp1*, Re*clp1*, and TU6-RP strains on different carbon sources and conidia formation on malt extract. **(B)** Quantitative determination of the colony diameters as shown in **(A)**. **(C)** Biomass accumulation of the TU6-RP and two independent transformants of Δ*clp1* cultured with 1% (w/v) glucose as the sole carbon source. **(D)** Counting of conidia formed by Δ*clp1*, Re*clp1*, and TU6-RP strains with a hemocytometer after cultivation on malt extract agar for 5 days. Significant difference (*t* test, *p < 0.05, **p < 0.01) was observed in colony diameter and conidia formation between the TU6-RP and the Δ*clp1* strains. Values represent the mean of two to three biological replicates. Error bars are the SD from these replicates.

To figure out the physiological role of *clp1* in *T. reesei*, the *clp1* null mutant was generated by specifically deleting its coding sequence, and its phenotypes in growth and conidiation were investigated. As shown in Figures 2A,B, the hyphal growth of the *clp1* null mutant on agar plates with different carbon sources was all slightly compromised after incubation at 30°C for 2 days. However, the growth rate of the mutant strain is comparable to that of the control strain when cultured in liquid MA medium in the presence of glucose with roughly equivalent final biomass (Figure 2C). In contrast with the hyphal growth, the Δ*clp1* mutant exhibited a severe defect in sporulation on malt extract agar plate (Figure 2A), as demonstrated by the dramatic decrease in conidia formation as compared with the control strain (Figure 2D). The defect of the mutant strain in both growth and conidiation was rescued by reintroduction of a *clp1* expression cassette under the control of the *A. nidulans gpd* promoter (Figures 2A,D). Taken together, these results demonstrate that CLP1 plays an important physiological role in *T. reesei*, especially in asexual conidia formation.

### Deletion of *clp1* Compromised *T. reesei* Cellulase Production

**Figure 3:**
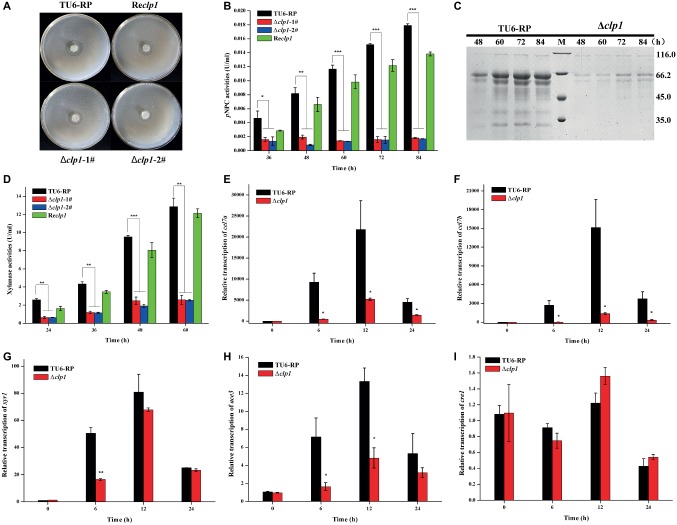
Absence of CLP1 compromises induced (hemi)cellulase gene expression. **(A)** Hydrolytic zone formation of Δ*clp1*, Re*clp1*, and TU6-RP strains on solid plate covered with a layer of 0.4% (w/v) ground Avicel. **(B)** Extracellular *p*NPC hydrolytic activities of the culture supernatant from Δ*clp1*, Re*clp1*, and TU6-RP strains cultured on 1% (w/v) Avicel. **(C)** SDS-PAGE analysis of the culture supernatant from TU6-RP and Δ*clp1* strains cultured on 1% (w/v) Avicel. **(D)** Extracellular xylanase activities of the culture supernatant from Δ*clp1*, Re*clp1*, and TU6-RP strains cultured on 0.5% (w/v) xylan. **(E–I)** Analyses of relative transcriptional levels of *cel7a* **(E)**, *cel7b* **(F)**, *xyr1* **(G)**, *ace3* **(H)**, and *cre1* **(I)** in the Δ*clp1* and TU6-RP strains cultured on 1% (w/v) Avicel using quantitative RT-PCR. Significant differences (*t* test, *p < 0.05, **p < 0.01, ***p < 0.001) were observed in the phenotypes as indicated between the control strain TU6-RP and the Δ*clp1* strain. Values represent the mean of three biological replicates. Error bars are the SD from these replicates.

To see the effect of the absence of CLP1 on cellulase production, the Δ*clp1* and the control strains were simultaneously inoculated on solid medium covered with a layer of 0.4% (w/v) ground Avicel. After incubation at 30°C for 5 days, Δ*clp1* colonies exhibited a much smaller hydrolytic zone than the control cells (Figure 3A), suggesting that *clp1* deletion impaired cellulase synthesis. To verify this result, the mutant and control strains were cultured in MA liquid medium with 1% (w/v) Avicel as the sole carbon source, and the extracellular cellulase activities were determined. As shown in Figure 3B, the Δ*clp1* strain exhibited a significant defect in extracellular *p*NPC hydrolytic activities compared to the control strain, which was largely rescued in the Re*clp1* strain (Figure 3B). SDS-PAGE analysis of the extracellular secreted proteins also showed a dramatic reduction in intensity for all corresponding bands in the mutant compared to those displayed by control strain (Figure 3C). The *clp1* deletion also caused a drastic reduction in the extracellular xylanase activity in the Δ*clp1* mutant compared to the control strain, which was largely rescued in the Re*clp1* strain when cultivated with xylan (Figure 3D). Further quantitative RT-PCR analyses indicated that the relative transcriptional expression of the two main cellulase genes *cel7a* and *cel7b* was dramatically decreased in Δ*clp1* (Figures 3E,F), demonstrating that the defective cellulase production resulting from *clp1* deletion occurred at the transcriptional level. To see the effect of the absence of CLP1 on other cellulase regulators, their endogenous transcripts were also determined (Figures 3G–I). The results showed that, whereas the transcription of *ace3* was significantly decreased on Avicel induction, the expression of *xyr1* was affected only at an early point of induction but remained comparable to the control strain later after. The expression of *cre1* was hardly affected in the absence of CLP1 compared with that of the control strain. Together, the data indicate that CLP1 plays an important role in regulating (hemi)cellulase production.

### The CLP1 Plant Homeo Domain Is Essential for *T. reesei* Cellulase Production and Conidiation

**Figure 4:**
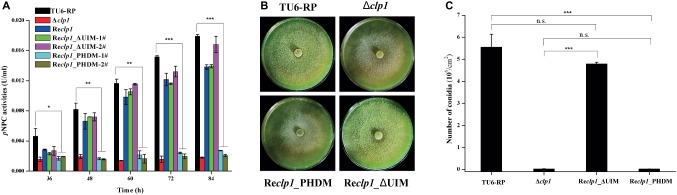
The integrity of the PHD is required for cellulase expression and conidiation. **(A)** Extracellular *p*NPC hydrolytic activities of the culture supernatant from the control strain TU6-RP, and the recombinant strains Δ*clp1*, Re*clp1*, Re*clp1*_PHDM, and Re*clp1*_ΔUIM cultured on 1% (w/v) Avicel. **(B)** Conidia formation of the control strain TU6-RP, and the recombinant strains Δ*clp1*, Re*clp1*, Re*clp1*_PHDM, and Re*clp1*_ΔUIM on malt extract agar plates at 30°C for 5 days. **(C)** Counting of conidia produced by the strains in **(B)**. Significant differences (*t* test, *p < 0.05, **p < 0.01, ***p < 0.001) were observed in (hemi)cellulase production and conidiation. Values represent the mean of three biological replicates. Error bars are the SD from these replicates.

To ascertain whether the PHD finger or the UIM in the CLP1 protein is important for the above-observed phenotypes, site-directed mutagenesis toward the PHD to disrupt its integrity or deletion of the UIM was performed. The cysteine residue 145 and histidine residue 150 that were predicted to be zinc ligands (Puig et al., 2004; Sanchez and Zhou, 2011) were simultaneously replaced by alanine to construct the CLP1_PDHM mutant while the UIM was deleted to generate the CLP1_ΔUIM mutant. These CLP1 mutants were individually expressed under the control of the *gpd* promoter in Δ*clp1* to generate the Re*clp1*_PDHM and Re*clp1*_ΔUIM strains, respectively. While UIM deletion hardly affected the extracellular cellulase activity and conidiation, the double point mutation of the PHD was unable to complement the Δ*clp1* conidiation and cellulase production defects (Figures 4A–C). These results strongly suggest that the integrity of the CLP1 PHD finger is important for its function in cellulase synthesis and asexual conidia formation.

### CLP1 Is a Nuclear Protein and Recruited to the Cellulase Gene Promoter Upon Cellulose Induction

**Figure 5:**
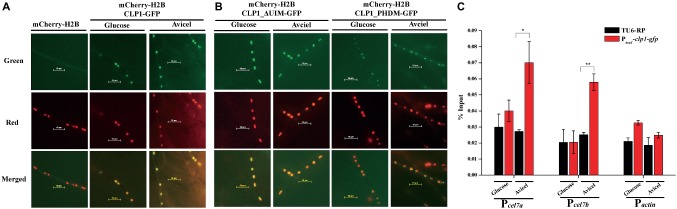
Subcellular localization of CLP1 and its mutants. **(A,B)** Subcellular localization of CLP1, the UIM-deleted mutant, and the PHD mutant with two point mutations (C145A and H150A) tagged with GFP at the C-terminus, respectively. Germinated hypha of *T. reesei* transformants of *mCherry*-*h2b*, *mCherry*-*h2b*&*clp1*-*gfp*, *mCherry*-*h2b*&*clp1_*ΔUIM-*gfp*, and *mCherry*-*h2b*&*clp1_*PHDM-*gfp* cultured on 1% (w/v) glucose or Avicel was subject to fluorescence analyses, respectively. **(C)** CLP1 is recruited to the cellulase gene promoters upon cellulose induction. ChIP analyses of CLP1 occupancy on the cellulase gene promoters of *cel7a*, *cel7b*, and the control gene *actin* in the P*_tcu1_*-*clp1*-*gfp* and TU6-RP strains cultured on 1% (w/v) Avicel or glucose for 24 h. Significant differences (*t* test, **p < 0.01, *p < 0.05) were observed in the occupancy of CLP1 on the *cel7a* and *cel7b* promoters between the control TU6-RP and the P*_tcu1_*-*clp1*-*gfp* strain under Avicel-inducing conditions. Values represent the mean of three biological replicates. Error bars are the SD from these replicates.

Given that the absence of CLP1 markedly impaired the transcription of cellulase genes, and that yeast CTI6 acts as a nuclear protein to mediate recruitment of specific regulators to the targeted promoters, we determined the subcellular localization of CLP1 in *T. reesei*. CLP1 epitope-tagged at the C-terminus with green fluorescent protein (GFP) was expressed in *T. reesei* TU-6 cells simultaneously expressing histone H2B that was epitope-tagged with the mCherry at the N-terminus. Fluorescence microscopic analysis showed that the green fluorescent signals from CLP1-GFP merged well with the red fluorescent signals from the mCherry-H2B, regardless whether the recombinant *T. reesei* cells were cultivated under the non-inducing (glucose) or the inducing (cellulose) condition (Figure 5A), demonstrating that CLP1 is a nuclear protein. Mutagenesis of amino acid residues 145 and 150 (C145 and H150 mutant CLP1_PDHM) or removal of the UIM (mutant CLP1_ΔUIM) did not affect CLP1-GFP nuclear localization. However, unlike CLP1_ΔUIM, CLP1_PDHM-GFP displayed an irregular pattern of accumulation in the nucleus (Figure 5B), suggesting that the integrity of the PHD finger contributes to the appropriate subcellular localization and function of CLP1 in the processes of cellulase production and conidiation.

To gain further insight into whether CLP1 is directly involved in the transcriptional regulation of cellulase genes, we expressed GFP-tagged CLP1 in Δ*clp1*, which was then subject to chromatin immunoprecipitation (ChIP) assays to determine CLP1 occupancy on cellulase gene promoters. As shown in Figure 5C, a significant enrichment of CLP1 was observed in the promoter regions of two main cellulase encoding genes, *cel7a* and *cel7b*, compared with the control cells that were both cultured under Avicel conditions. However, no such enrichment was observed under glucose conditions (Figure 5C), suggesting that CLP1 is recruited to the cellulase gene promoters specifically upon cellulose induction to be involved in cellulase gene activation.

### Overexpression of XYR1 Fully Rescued the Defect in Cellulase Production but Not Conidiation Resulting From *clp1* Deletion

**Figure 6:**
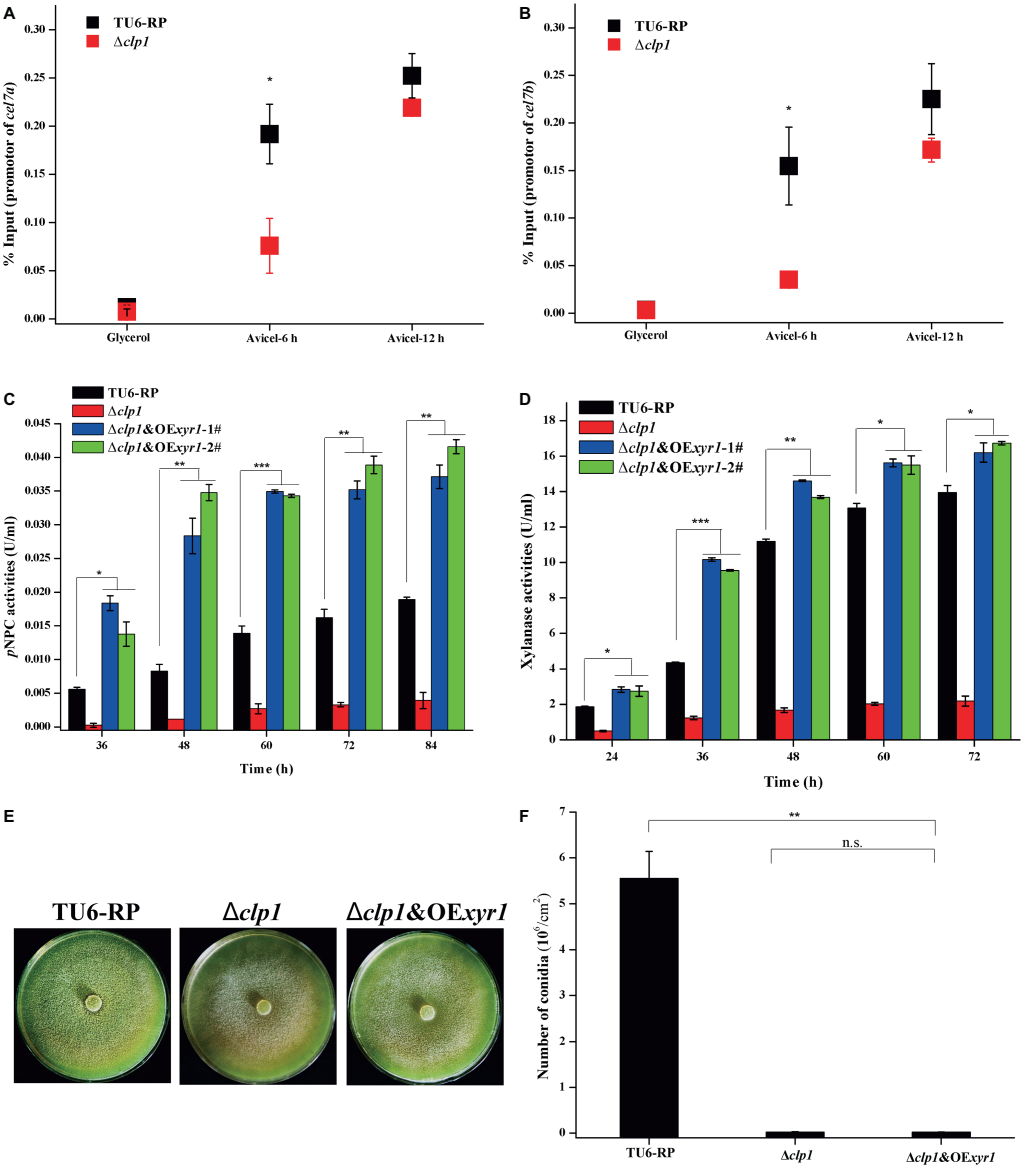
Overexpression of XYR1 fully rescued the defect in cellulase production but not conidiation resulting from *clp1* deletion. **(A,B)** ChIP analyses of XYR1 occupancy on *cel7a* **(A)** and *cel7b* **(B)** promoters in the Δ*clp1* and TU6-RP strains after cultivation on 1% (v/v) glycerol or 1% (w/v) Avicel for 6 and 12 h. **(C,D)** Extracellular *p*NPC hydrolytic activities **(C)** and xylanase activities **(D)** of the culture supernatant from TU6-RP, Δ*clp1*, and Δ*clp1*&OE*xyr1* strains cultured on 1% (w/v) Avicel or 0.5% (w/v) xylan. **(E,F)** Conidia formation of the control strain TU6-RP, Δ*clp1*, and Δ*clp1*&OE*xyr1* strains incubated on malt extract agar plates at 30°C for 5 days **(E)** and conidia counting **(F)**. Significant differences (*t* test, *p < 0.05, **p < 0.01, ***p < 0.001) were observed in the (hemi)cellulase production and conidiation between the control strain TU6-RP and the Δ*clp1*&OE*xyr1* strain. Values represent the mean of three biological replicates. Error bars are the SD from these replicates.

As described above, *clp1* mutants are defective for the induced transcription of *ace3* while *xyr1* transcription was also decreased at an early stage of induction. Both genes have been reported to encode key transactivators in regulating *T. reesei* cellulase gene expression (Stricker et al., 2006; Ma et al., 2016). We determined XYR1 occupancy on cellulase gene promoters in both TU6-RP and Δ*clp1* cells using ChIP analyses (Figures 6A,B). In contrast with control cells wherein a marked increase in XYR1 binding to cellulase gene promoters was observed when the carbon source was shifted from glycerol to Avicel, XYR1 binding was compromised in Δ*clp1* cells only during the early cultivation on Avicel, which is consistent with the pattern of *xyr1* transcription. We next overexpressed ACE3 or XYR1 using the *tcu1* promoter in Δ*clp1*, to investigate whether sufficient amounts of ACE3 or XYR1 would rescue the defect in (hemi)cellulase production in the *clp1* mutant. While ACE3 overexpression had no effect on the defective cellulase gene expression (Supplementary Figure S2), XYR1 overexpression fully restored the induced cellulase and xylanase production in Δ*clp1* (Figures 6C,D). XYR1 overexpression, however, had hardly any effect on the compromised conidia formation in Δ*clp1* (Figures 6E,F). These results suggest that CLP1 participates in modulating cellulase gene expression not only by facilitating either *xyr1* expression or XYR1 binding to cellulase gene promoters, but also may contribute to the transcriptional activation process mediated by XYR1.

## Discussion

*T. reesei* is one of the most important cellulase-producing fungi applied in industry. To identify more regulators involved in *T. reesei* cellulase gene expression, we performed a blast analysis in its genome with the sequence of CTI6, which is a novel PHD protein involved in activating or repressing a number of genes in *S. cerevisiae via* mediating the recruitment of chromatin modifiers to the target gene promoter regions (Papamichos-Chronakis et al., 2002; Wang et al., 2011). This analysis retrieved the CLP1 protein sharing quite low sequence identity (19%) and similarity (29%) with CTI6. Whereas it is still arbitrary to assign CLP1 as a CTI6 homolog in *T. reesei* due to their low sequence identity, homologs of CLP1 are widely distributed in filamentous fungi including *Trichoderma*, *Fusarium*, *Neurospora*, *Penicillium*, and *Aspergillus* species, sharing relatively higher sequence identity (30–98%) with CLP1, although none of these homologs have been characterized.

We presented evidence that CLP1 is an important positive regulator of the induced cellulase gene expression in *T. reesei*. Similar to CTI6 and other transcriptional regulators, CLP1 is targeted to the nucleus although it does not have the obvious nucleus localization sequence (NLS). An explanation for this anomaly may be that CLP1 is targeted to the nucleus with the help of other as-yet-unknown partners with NLS by direct or indirect interactions. Moreover, ChIP analyses demonstrated that CLP1 is recruited to promoters of the main cellulase genes specifically upon cellulose induction, further supporting that CLP1 directly regulates cellulase gene expression. In addition, CLP1 is also found to be functional in conidia formation, which is an independent physiological process from cellulase expression, suggesting that CLP1 simultaneously exerts its regulatory effect on genes involved in sporulation. In *A. nidulans*, activation of *brlA* expression is an essential step of conidiation (Adams et al., 1988). Knockout of *brlA* results in a bristle-like structure that produces an elongated stalk and fails to develop vesicles or any other subsequent structures (Clutterbuck, 1969; Boylan et al., 1987). By contrast, overexpression of *brlA* leads to the formation of viable conidia directly from the hyphal tips (Adams et al., 1988). Although the homologous protein of BRLA has not been found in *T. reesei*, several other regulators including LAE1 and VEL1 that participate in modulating sporulation and cellulase production have been identified (Seiboth et al., 2012; Karimi Aghcheh et al., 2014). However, the detailed genetic relationship between CLP1 and these regulators in controlling sporulation warrants further study.

CLP1 contains a conserved PHD finger (~60 aa), which is also observed in CTI6, although its role in CTI6 function has not been clarified. The PHD finger occurs in a set of proteins, the majority of which are involved in chromatin-mediated transcriptional regulation either directly or through protein-protein interactions (Aasland et al., 1995; Schultz et al., 2001). However, evidence exist that the PHD is dispensable for specific interactions with transcriptional regulatory complexes including GCN5/SAGA but is essential for CTI6 function at the *GAL1* promoter (Papamichos-Chronakis et al., 2002). On the other hand, the integrity of CTI6 PHD finger is not required for its targeting to the nucleus (Puig et al., 2004). In accordance with these results, our data showed that the integrity of CLP1 PHD finger is essential for its regulatory activity in cellulase expression and conidia formation, as demonstrated by the observation that the double point mutant in PHD is similarly defective for cellulase synthesis and conidiation. However, in contrast with the normal nuclear localization of CTI6 PHD mutant in *S. cerevisiae* (Puig et al., 2004), CLP1_PDHM displayed a subtly different pattern of nuclear accumulation compared with WT CLP1. One could speculate that the malfunction of the *clp1* double point mutant resulted from its abnormal nuclear distribution. In contrast with the PHD, removal of the UIM from CLP1, which is present in homologs of most filamentous fungi but not in *S. cerevisiae* CTI6, did not influence the cellular localization and function of CLP1. The function of UIM in CLP1 as well as its fungal homologs awaits further investigation. Nonetheless, these mutational analyses suggested that *T. reesei* CLP1 may adopt a quite different folded structure from *S. cerevisiae* CTI6 and thus CLP1 probably represents a novel transcriptional regulator.

XYR1 is the dominating transcriptional activator in *T. reesei* cellulase gene expression. While the absence of XYR1 abolished almost all the cellulase and hemicellulase gene expression (Stricker et al., 2006; Ma et al., 2016), its overexpression results in a full expression of cellulases even under non-inducing conditions (Lv et al., 2015). Although XYR1 occupancy on cellulase gene promoters as well as its transcriptional expression was compromised in the *clp1* null mutant at the early cultivation phase on cellulose (6 h), it should be noticed that both were comparable to those in the control strain when the cultivation period was extended to 12 h (Figures 3B,G, 6A). Thus, the defective cellulase gene expression during the whole inducing process cannot be solely accounted for by the decreased XYR1 binding to cellulase gene promoters at the early stage of induction. Possibility exists that CLP1 either contributes to XYR1-mediated transcriptional activity when recruited to promoters or just acts in parallel with XYR1 in the transcriptional activation process. The observation that XYR1 overexpression fully rescued the defect in cellulase production but not conidiation in *clp1* mutant further supports the above note that excessive XYR1 may override the requirement for CLP1. Unlike XYR1, ACE3 overexpression is not able to rescue the defective cellulase production in Δ*clp1*. This observation is supported by the observation that *ace3* expression is not upregulated with XYR1 overexpression, which leads to full cellulase gene expression under non-inducing conditions (data not shown). Considering that CLP1 contains the PHD finger that is commonly observed in proteins that may participate in modifying chromatin as well as mediating molecular interactions in gene transcription (Aasland et al., 1995; Sanchez and Zhou, 2011), we speculate that CLP1 acts as a cofactor to contribute to recruiting unknown factors such as chromatin modifiers or remodelers playing important roles in the process of assembling transcriptional machinery and thus activation of cellulase genes.

## Data Availability

The raw data supporting the conclusions of this manuscript will be made available by the authors, without undue reservation, to any qualified researcher.

## Ethics Statement

No human studies are presented in this manuscript. No animal studies are presented in this manuscript. No potentially identifiable human images or data is presented in this study.

## Author Contributions

LW and RY performed the experiments. YC, FZ, XM, YZ, and GC performed data analysis. WL, WZ, and WL designed the project. WL, WZ, and LW wrote the manuscript.

### Conflict of Interest Statement

The authors declare that the research was conducted in the absence of any commercial or financial relationships that could be construed as a potential conflict of interest.

## Footnotes

^1^https://www.uniprot.org/
