# Supplementary material for: CLP1, a Novel Plant Homeo Domain Protein, Participates in Regulating Cellulase Gene Expression in the Filamentous Fungus Trichoderma reesei
Source: Front Microbiol. 2019 Aug 6;10:1700. doi: 10.3389/fmicb.2019.01700 (PMC6691364; doi:10.3389/fmicb.2019.01700)
Supplement: Supplementary file 1 [file Data_Sheet_1.PDF]

CLP1, a novel PHD domain protein, participates in regulating cellulase gene expression in the filamentous fungus *Trichoderma reesei*

Lei Wang, Renfei Yang, Yanli Cao, Fanglin Zheng, Xiangfeng Meng, Guanjun Chen, Weixin Zhang*, Weifeng Liu*

State Key Laboratory of Microbial Technology, Shandong University, No.72 Binhai Road, Qingdao 266237, P. R. China

*Correspondence should be addressed to W Zhang or W Liu.

E-mail: zhangwx@sdu.edu.cn; weifliu@sdu.edu.cn

Supplemental Data

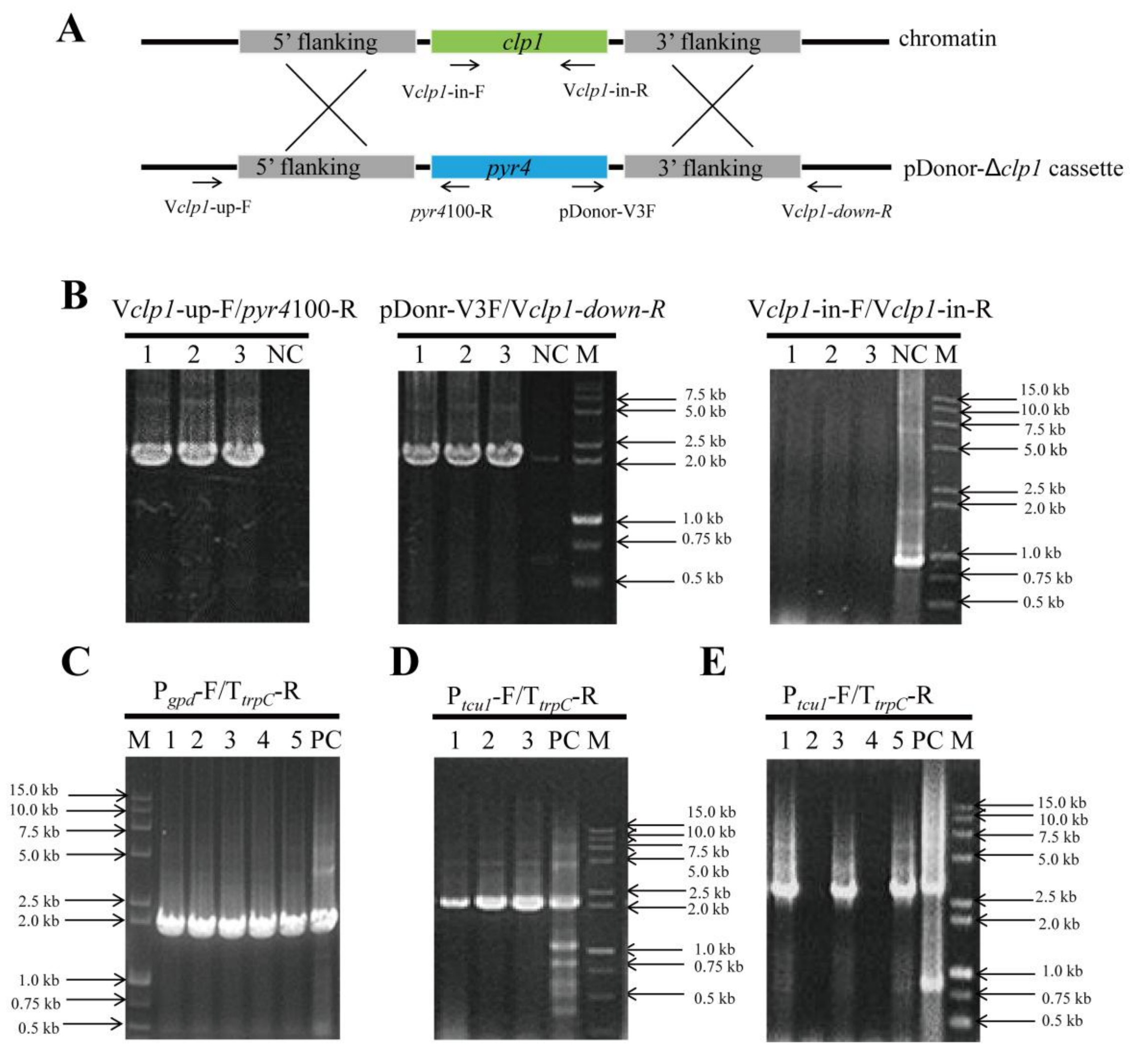

Fig. S1. Verification of correct DNA integration in the *T. reesei* mutant strains constructed in this study. (A-B) Schematic illustration of the *clp1* deletion cassette (A) and verification for its correct integration into the genome (B). (C) PCR amplification to verify correct integration of P_{gpd}-*clp1*-T_{trpC}, P_{gpd}-*clp1*_ΔUIM-T_{trpC} and P_{gpd}-*clp1*_PHDM-T_{trpC} plasmids in the Δ*clp1* strain, respectively. (D) PCR amplification to verify correct integration of pMDP_{tcu1}-*ace3*-T_{trpC} plasmid in the Δ*clp1*&OE*ace3* strain. (E) PCR amplification to verify correct integration of

pMDP$_{tcu1}$-*xyr1*-T$_{trpC}$ plasmid in the Δ*clp1*&OE*xyr1* strain. All the amplified fragments correspond to the expected size.

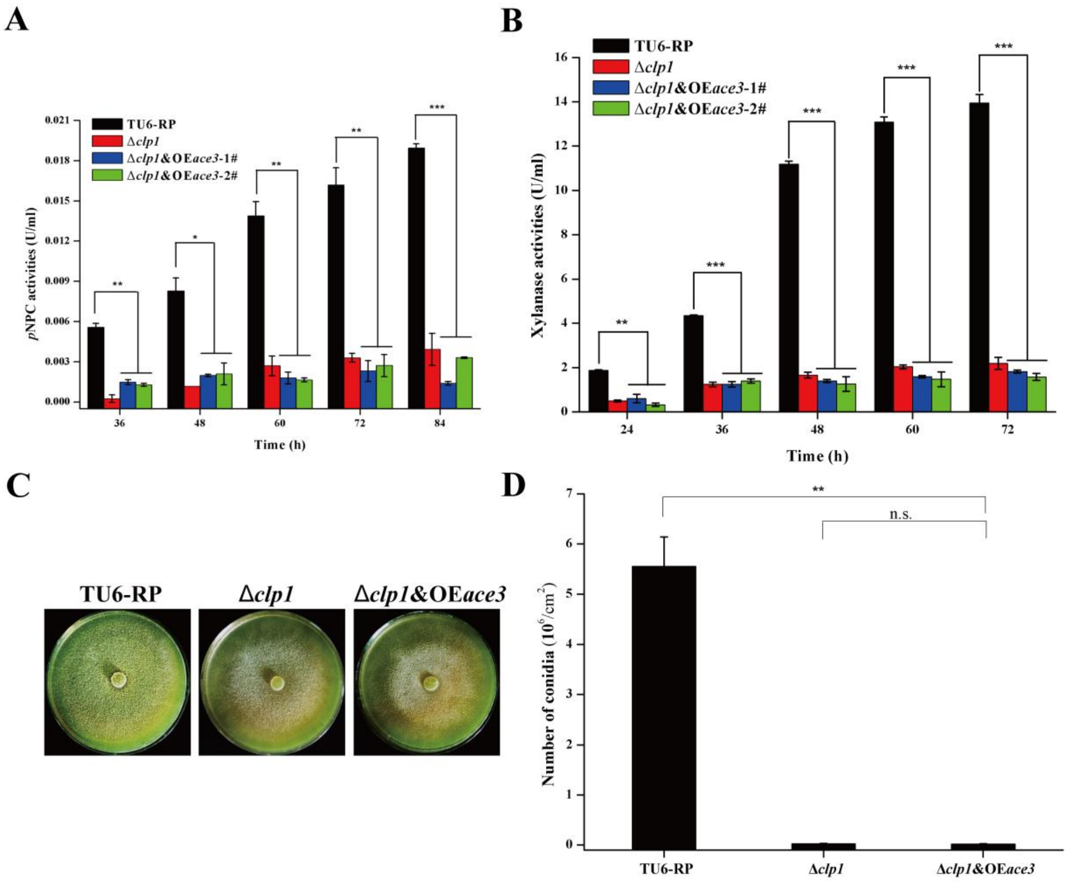

Fig. S2. Overexpression of ACE3 did not rescue the defect of cellulase expression and conidiation resulted from *clp1* deletion. (A-B) Extracellular *p*NPC activities (A) and xylanase activities (B) of the culture supernatant from TU6-RP, Δ*clp1* and Δ*clp1*&OE*ace3* strains cultured on 1% (w/v) Avicel or 0.5% (w/v) xylan. (C-D) Conidia formation of the control strain TU6-RP, and Δ*clp1* and Δ*clp1*&OE*xyr1* incubated on malt extract agar plates at 30 ℃ for 5 days (E) and conidia counting (F).
